# Focused reservoir characterization: analysis of selected sand units using well log and 3-D seismic data in 'Kukih' field, Onshore Niger Delta, Nigeria

**DOI:** 10.1038/s41598-024-56100-7

**Published:** 2024-06-14

**Authors:** Olufisayo Ibukun Fagbemi, Abel Idowu Olayinka, Michael Adeyinka Oladunjoye, Paul Irikefe Edigbue

**Affiliations:** 1 Department of Geology, University of Ibadan, Ibadan, Oyo State Nigeria; 2 King Fahd University of Petroleum and Minerals, Dhahran, Saudi Arabia

**Keywords:** Reservoir characterization, Hydrocarbon potential, Depositional environment, Petrophysical evaluation, Structural analysis, Facies analysis, Multidisciplinary approach, Geology, Geophysics, Sedimentology, Seismology

## Abstract

This study focuses on the comprehensive reservoir characterization of the ‘Kukih’ Field within the onshore northeastern Niger Delta region, Nigeria. The absence of its detailed description with delineated reservoir properties, lateral continuity, and their use to identify potential reservoir quality and heterogeneity necessitated this study. Integrating well log and 3D seismic data, the investigation aims to elucidate reservoir properties, lithofacies, and depositional environments to unravel hydrocarbon potential. The geological setting, encompassing the Agbada Formation of Early and Middle Miocene age, is scrutinized through detailed geologic analysis. Petrophysical evaluation of four well logs (Kukih-1, Kukih-2, Kukih-3, and Kukih-4) facilitated the determination of key parameters such as shale volume, effective porosity, and water saturation. Seismic interpretation further enriched the structural characterization of the field. Results showcase three predominant reservoir sands (A, B, and C) with distinct lithofacies and thickness variations. Effective porosity ranges from Fair to Excellent, with permeability exhibiting high values for hydrocarbon reservoir potential. Water saturation trends, lithofacies distributions, and structural features were illuminated through iso-parametric maps and seismic analyses. Depositional environments were inferred through facies analysis, revealing the presence of funnel-, cylinder-, and bell-shaped successions that hint at intricate marine sedimentary processes. Challenges owing to limited core data were acknowledged, and the integration of methodologies emerged as a pivotal strategy for enhanced reservoir understanding. This study underscores the ‘Kukih’ Field's hydrocarbon potential, accentuating the significance of multidisciplinary approaches in deciphering complex reservoir systems. In light of the petrophysical analysis derived from the well logs and the identification of structural highs through the structural maps, this study recommends the drilling of unexplored zones exhibiting promising structural characteristics.

## Introduction

The success of hydrocarbon exploration within a given field hinges upon the meticulous and precise assessment of formations. This includes the identification of hydrocarbon resources, a comprehensive analysis of formation fluids, and a foundation built upon the utilization of high-quality data. While the evaluation of formations and the determination of reservoir performance present inherent challenges^1^, the delineation of critical petrophysical parameters such as shale volume, porosity, permeability, and water saturation plays a pivotal role in understanding reservoir capabilities^2^.

Although the limited number of wells in Kukih Field and the absence of core data proved a challenge, the justification for more wells needs to be established by exploring the potential of the existing ones and determining the reservoir’s lateral continuity and penetration depth. Due to the observed range of complexities in the depositional settings of the area caused by its petrophysical and sedimentological characteristics, the integrated use of well logs for petrophysical parameter derivation and cross plots analysis founded on the solid application of depositional environmental trend analysis can provide a reliable delineation of reservoir flows in Kukih Field even with the unavailability of core samples.

The seamless integration of robust and pertinent data, in conjunction with a firm grasp of the geological aspects of the formation, such as its depositional history, serves as the cornerstone for defining depositional environments and achieving an effective reservoir characterization^3^. Within the scope of this study, the primary objectives encompass the identification of distinct lithological units, and a comprehensive evaluation of petrophysical and lithological properties intrinsic to the reservoir sand involving average shale volume, effective porosity, and water saturation. Additionally, this study seeks to ascertain the distribution and lateral continuity of reservoir properties, while also delineating the structural attributes and potential hydrocarbon-bearing sands inherent to the field.

The novelty of this study lies in its holistic approach to reservoir characterization in the Kukih Field, combining advanced petrophysical analyses with 3D seismic data interpretation. This comprehensive integration not only unveils the hydrocarbon potential of known reservoirs but also identifies previously untested structural zones, offering a fresh perspective on potential hydrocarbon accumulations. Furthermore, the study delves into the depositional environments of these reservoirs, illuminating their sedimentary history essential for hydrocarbon extraction. However, it does not detail its sequence stratigraphy, nor account for the lateral and vertical relationships of rock layers to ascertain the historical and environmental context of rock formation and geological processes. Such an integrated methodology, including multiple aspects of reservoir evaluation and recommendations for further studies, provides an adequate yet novel framework for decision-making in hydrocarbon exploration, optimizing resource utilization and reducing uncertainty in the field's development^4^.

## Geological context of the study area

**Figure 1:**
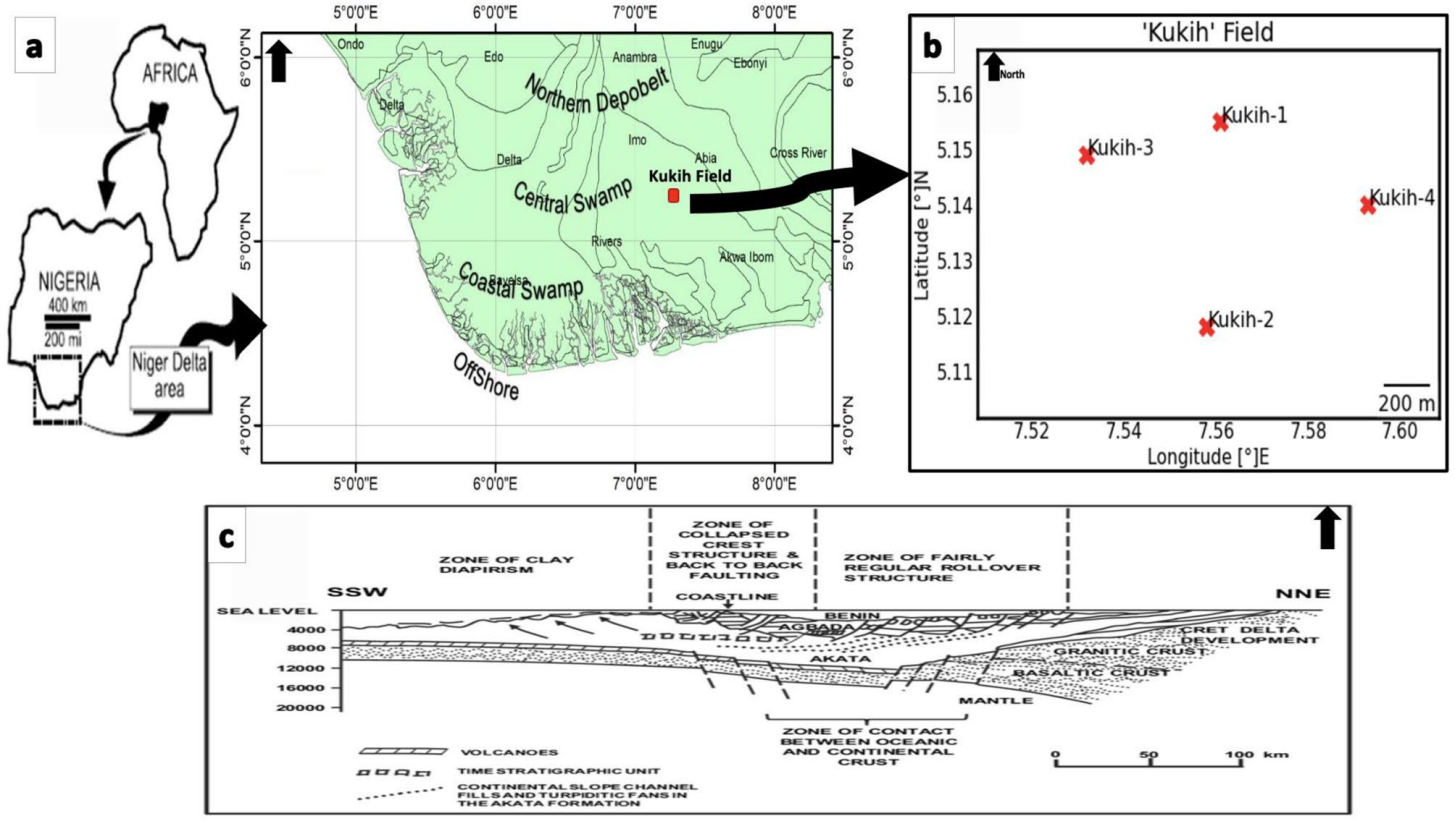
Showing (**a**) Kukih Field in relation to the Niger Delta, along the West Coast of Africa, with structures of depobelts, after ^6,9^. (**b**) Location Map of the Kukih Field with the well locations. Self-developed from well log data with Python [v3.8.8] Programming Language from the Jupyter Notebook [v6.3.0], a web-based, interactive computing platform. (**c**) Diagrammatic dip profile of the Niger Delta, after ^5^.

The Kukih Field occupies an onshore position within the northeastern expanse of the Niger Delta region of the Greater Ughelli depobelt, encompassing the Early and Middle Miocene epochs of the Agbada Formation^5,6^. This locale is geographically situated between Longitudes 7.53°E and 7.59°E, as well as Latitudes 5.13°N and 5.54°N, as substantiated by^7^. The geographical depiction of the Kukih Field and the wells at hand is illustrated in Fig. 1. The Tertiary Niger Delta occupies a strategic position within the continental margin of the Gulf of Guinea, covering an expansive area of more than 75,000 square kilometers. This entity materializes as a regressive clastic sequence characterized by a substantial thickness surpassing 9,000 units. Noteworthy for its prominence, it stands as the preeminent inland basin within West Africa. Its confines are delineated by the Okitipupa basement high to the west, demarcating its separation from the Dahomey Basin, also known as the Benin Basin. To the east, its boundaries coincide with the Cameroun volcanic line. Within the overview of geological landscapes, the Nigerian Basin emerges as an integral fragment of the expansive Benue Trough. This elongated intracontinental basin extends its reach from the western extremities of the Gulf of Guinea to the eastern frontiers of Sudan. The Benue Trough emerged as a cardinal geological formation within the West African context with origins traced back to the Mesozoic era's supercontinent fragmentation. This geological marvel is traversed by various ancillary elements in the northern quadrant, including the Abakaliki Basin, the Calabar Flank, and the Anambra Basin^8^.

The geological evolution of the Niger Delta basin is intricately influenced by both pre- and syn-sedimentary tectonic activities, as elucidated by^10–12^. Preceding sedimentation, Cretaceous Fracture zones were engendered through pre-sedimentary tectonic activities, manifesting prominently as trenches and ridges within the deep Atlantic. Concurrent with sedimentation, syn-sedimentary tectonic activities, notably gravity tectonics, came into play, exhibiting activity subsequent to the rifting episode (Fig. 1c). The manifestations of these gravity tectonic events include the formation of synthetic and antithetic growth faults, roll-over anticlines, and salt diapirs, contributing significantly to the internal structural configuration of the basin.

The Tertiary sedimentary deposits within the Niger Delta Basin are characterized by the sequential accumulation of three major stratigraphic units^6^. The basal sequence comprises consistently developed dark grey shale known as the Akata Formation, ranging in age from Eocene to Recent. Overlying this is a predominant marine sand-shale succession, recognized as the Agbada Formation, which constitutes the primary target for oil exploration in southern Nigeria. Hydrocarbon discoveries within the Agbada Formation span from the Eocene to potentially the Pliocene–Pleistocene period^13^. Finally, the youngest stratigraphic sequence, identified as the Benin Formation, is characterized by predominantly massive continental sand deposits, spanning in age from Eocene to Recent.

## Methodology employed in the study

The study methodology is structured around three primary datasets: well-log data, check shot data for time-depth relationship and seismic data. The comprehensive research materials are meticulously categorized to ensure a systematic analysis. Within the ambit of well-log data, the focus converges on four composite well-log suites namely, K-1, K-2, K-3, and K-4. These well-log suites, integral to the petrophysical evaluation of the Kukih Field, amalgamate diverse measurements encompassing gamma-ray (GR), resistivity (RES), neutron, density, and sonic logs. This multifaceted suite forms the cornerstone for analyzing and comprehending the petrophysical attributes of the Kukih Field. These logs play a fundamental role in estimating the field's petrophysical parameters, thereby facilitating a nuanced understanding of the depositional environment. The wells under scrutiny are characterized by varying drilling depths, spanning approximately 2246 m, 3038 m, 2472 m, and 50 m, respectively. The well-log dataset offers a comprehensive insight into the subsurface dynamics. Referencing Table 1, a concise representation of the wells and the corresponding available logs is delineated. It's noteworthy to mention that K-3 stands as a deviated well, characterized by its northward trajectory. Complementing the well-log analysis, the study extends its purview to seismic interpretation. This encompasses a holistic approach, incorporating a well-to-seismic tie process, generating time maps and depth maps, and culminating in the intricate task of reservoir static modelling. These endeavours are undertaken with the overarching aim of interpreting the prevalent facies within the geological formation.

**Table 1:** Wells and available wireline logs for this study.

Wells	TD*	GR	RES	Neutron	Density	Sonic	Dev. data	Check shot
K-1	2246	✓	✓	✓	✓	✓	✓	✓
K-2	3038	✓	✓	✘	✓	✘	✓	✘
K-3	2472	✓	✓	✘	✓	✘	✓	✘
K-4	50	✓	✓	✘	✘	✘	✓	✘

TD*: true depth (m), GR: gamma ray (API), and RES: resistivity (Ωm).

**Figure 2:**
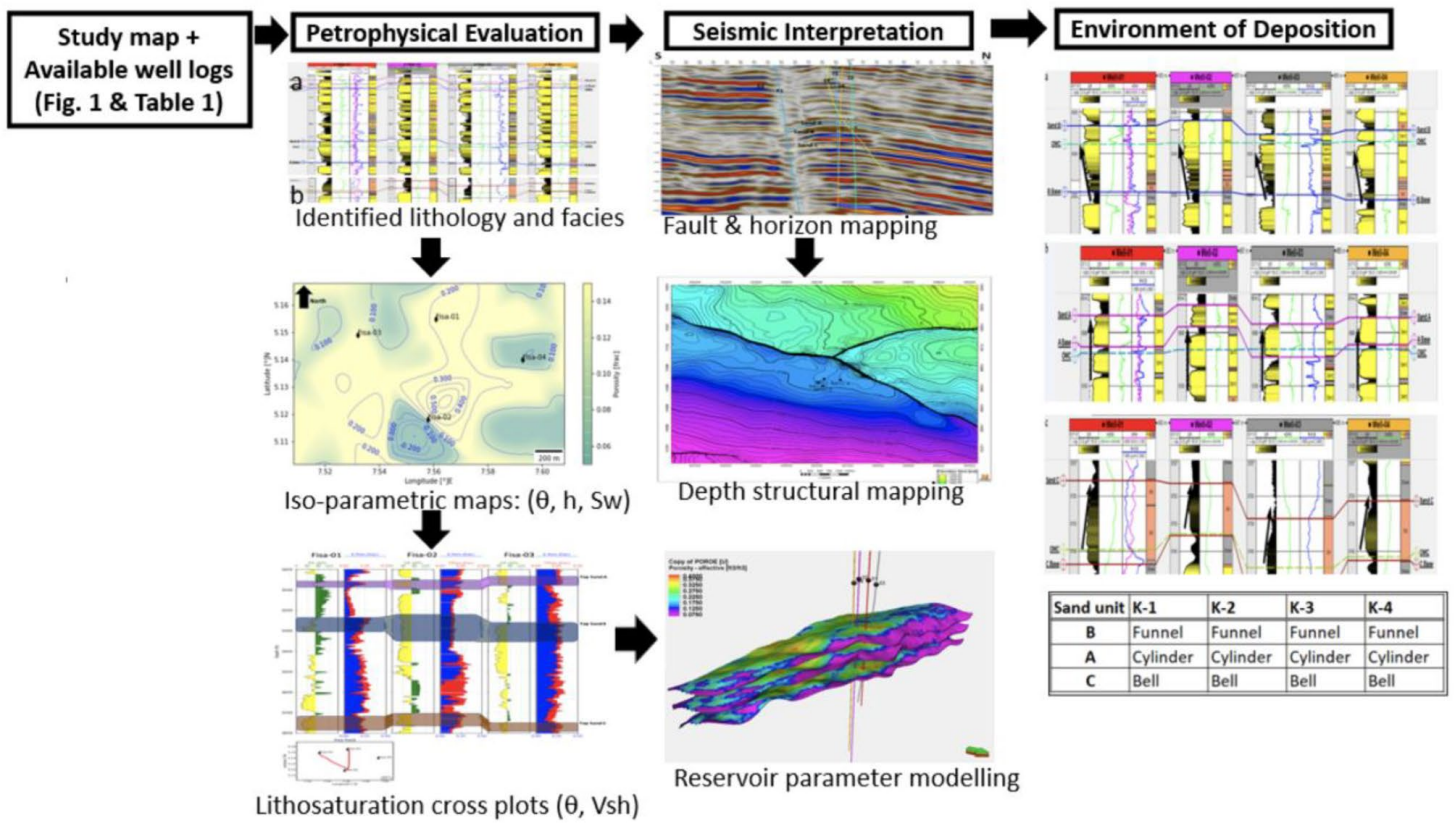
Methodological flowchart arranged from selected images from subsections of the study.

The study comprises three primary sections, as outlined in the methodological diagram (Fig. 2).

### Petrophysical assessment

Through the analysis of well logs, reservoirs have been meticulously identified, correlated, and spatially delineated across the well locations, prioritizing pertinent facies. The extraction of petrophysical parameters has facilitated the creation of parametric maps in accordance with the methodologies expounded by^14^. This inclusive approach enables the derivation of critical metrics including the Volume of Shale (${V}_{sh}$), Effective Porosity (${\theta }_{e}$), Water Saturation (${S}_{w}$), Net Thickness, and lithosaturation maps.

### Calculation of shale volume (${{\varvec{V}}}_{{\varvec{s}}{\varvec{h}}}$)

For the estimation of Shale Volume (${V}_{sh}$), the Gamma-ray technique has been judiciously applied in line with Eq. 1. It is imperative to underscore that within the Agbada Formation, a necessary correction for the shale effect has been undertaken, guided by the insights of^15^. This correction factor serves to fine-tune the accuracy of the derived results. The computation necessitates the incorporation of the minimum Gamma-ray (${GR}_{min}$), in addition to the determination of both the shale volume and the Gamma-ray Index (IGR).

1 $${I}_{GR}=\frac{({GR}_{log}-{GR}_{min})}{({GR}_{max}-{GR}_{min})}$$

where ${I}_{GR}$ is the volume of clay, ${V}_{sh}$ is the shale volume, ${GR}_{log}$ is the log reading at depth of interest, ${GR}_{max}$ is the value in a Shale of the Formation, ${GR}_{min}$ is the value at the clean sand zone of the Formation.

2 $${V}_{sh}=0.083\left({2}^{(3.7\times {I}_{GR})}-1\right)$$

Reservoir characterization based on ${V}_{sh}$ is shown in Table 2.

**Table 2:** ${V}_{sh}$ classification after ^3,16^.

${V}_{sh}$(%)	Classification (${V}_{sh}$)	Porosity (%)	Classification (${\theta }_{e}$)
< 5	Sand	< 5	Negligible
5–15	Moderately shaly sand	6–10	Poor
15–25	Shaly sand	11–15	Fair
25–35	Very shaly sand	16–20	Good
> 35	Shale	21–25	Very good
		> 25	Excellent

From Table 2, the portion of the reservoir having low ${V}_{sh}$ corresponds to a sandy reservoir, while the high ${V}_{sh}$ corresponds to a shally reservoir therefore, the lower the ${V}_{sh}$, the better the reservoir.

### Porosity (${{\varvec{\theta}}}_{{\varvec{t}}},\boldsymbol{ }{{\varvec{\theta}}}_{{\varvec{e}}}$)

3 $${\theta }_{t}=\frac{{\rho }_{ma}-{\rho }_{b}}{{\rho }_{ma}-{\rho }_{fl}}$$

where ${\rho }_{ma}$ is the sandstone’s grain (or matrix) density, ${\rho }_{b}$ is the bulk density, and ${\rho }_{fl}$ is the density of the fluid.

4 $${\theta }_{e}=\left(1-{V}_{sh}\right)\times {\theta }_{t}$$

where ${\theta }_{e}$ is the effective porosity, (${\rho }_{ma}$ = 2.65 g/cc, ${\rho }_{b}$ = 1.0 g/cc).

The effective porosity is relevant to reservoir characterization because the higher the porosity value, the better the reservoir^17^, as shown in Table 2.

### Water saturation (${{\varvec{S}}}_{{\varvec{w}}}$)

This is the amount of pore in a reservoir occupied by formation water. The water saturation is usually calculated with Archie’s equation.

5 $${S}_{w}=\sqrt[n]{(a.\frac{{R}_{w}}{{{R}_{t}* \varnothing }^{m}})}$$

where $m$ is the cementation factor (default value is 2.0), $n$ is the saturation exponent (default value is 2.0), ${R}_{w}$ is the formation water resistivity, and ${R}_{t}$ is the Formation’s true resistivity. The hydrocarbon saturation is estimated as;

6 $${S}_{h}=1-{S}_{w}$$

### Permeability (K)

Among many proposed empirical relationships with which permeability can be estimated from porosity, this study utilizes the empirical expression for permeability estimation by^18^; it estimates the intrinsic (absolute) permeability. This method will be more appropriate because it considers the unconsolidated reservoir sand within the Niger Delta hydrocarbon province. The empirical permeability for gas and oil are:

7 $$K={(79\times \frac{{\mathrm{\varnothing }}_{e}^{3}}{{S}_{w}})}^{2}\mathrm{ for\, Gas}$$

And,

8 $$K=26552\times {\mathrm{\varnothing }}_{e}^{2}-34540\times {\left({\mathrm{\varnothing }}_{e}\right)}^{2}+307 \mathrm{\,for\, Oil}$$

where $$K=Permeability (mD)$$, $${\mathrm{\varnothing }}_{e}=Effective\, porosity$$, $${S}_{w}=Water\, saturation$$

A reservoir at irreducible water saturation $\left({S}_{wirr}\right)$, water in the uninvaded zone $\left({S}_{w}\right)$ will be static, i.e., very close to constant, and hydrocarbon production at the irreducible water Saturation zone will be water free.

9 $${S}_{wirr}=0.677823-0.00735664\times \sqrt{\frac{K(mD)}{{\mathrm{\varnothing }}_{e}}}$$

where $${S}_{wirr}=Irreducible\, water\, saturation$$

#### Seismic interpretation

**Figure 3:**
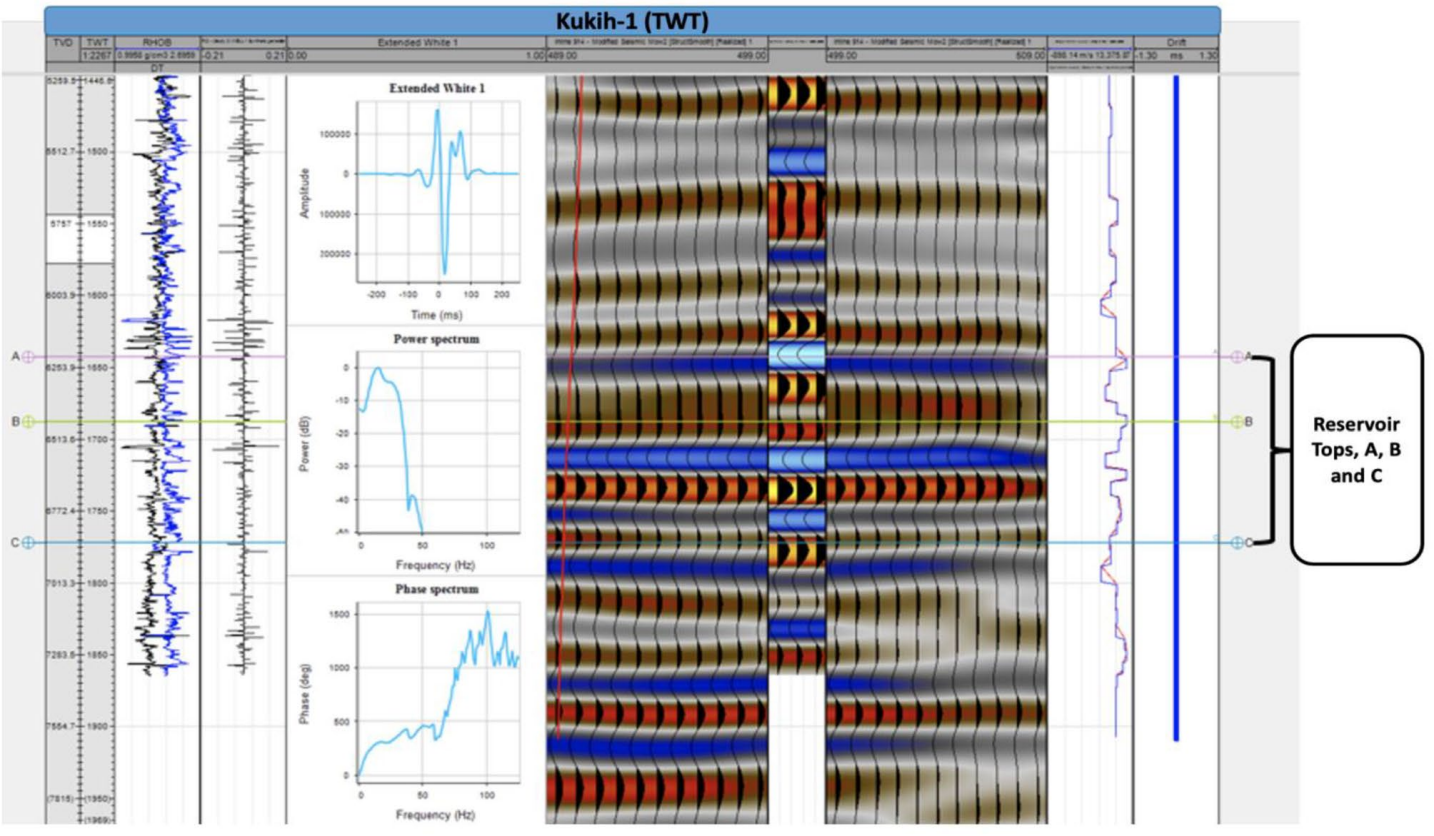
Workflow output of well-to-seismic tie for Kukih-1, with top horizons of prospect zones (A, B and C), True Vertical Depth TVD (ft), Two-way Time (TWT), Density-Sonic Cross plot, Reflectivity series, Power and Phase spectrum, Seismic traces, Synthetic Seismogram (inserted), Acoustic impedance and Drift (ms).

Incorporating 3D seismic data spanning Inline values from 740 to 1040 and Crossline values ranging from 280 to 680, a comprehensive analysis ensued to delineate the structural makeup of the field. Leveraging the reflectivity signals inherent to geologic events, both synthetic and antithetic faults were painstakingly mapped. This endeavour was facilitated by engaging in a seismic-to-well tie, and subsequently identifying the horizons of interest. To further enhance accuracy, time structure maps were meticulously generated utilizing the velocity information from the checkshot data. By aligning reservoir tops within the seismic sections and drawing upon the sonic log, the conversion of time maps into depth maps was accomplished and effectively calibrated through the synchronization of sonic and density logs^19,20^. The process of creating a synthetic seismogram entailed a rigorous calibration of well logs, coupled with the careful selection of reflectivity series. This was followed by the convolution of these reflectivity series with a seismic-source representative wavelet. The pursuit of an optimal match, achieved through waveform estimation, solidified the authenticity of the synthetic seismogram (Fig. 3). Notably, a seamless well-to-seismic tie was accomplished, characterized by the absence of bulk shifts, squeezes, or stretches.

Therefore, the endeavour culminated in a thorough attempt to reconstruct a 2D or 3D representation of the subsurface terrain, meticulously anchored upon structural, fault, facies, stratigraphic, and petrophysical attributes derived from reservoir interpretation. Probabilistic techniques supporting the facies model algorithm were employed to understand the static reservoir descriptions with dimension. This facilitated the development of reservoir parameters such as porosity and permeability^21^. Noteworthy is the derivation of water saturation models, intricately derived from the permeability model through cross-plot functions and statistical estimations derived from Sequential Gaussian Simulation (SGS) techniques. These methodologies were instrumental in delineating the lateral continuity of reservoir properties, considering the spacing of reservoir bodies from well sources.

#### Petrophysical modelling

**Figure 4:**
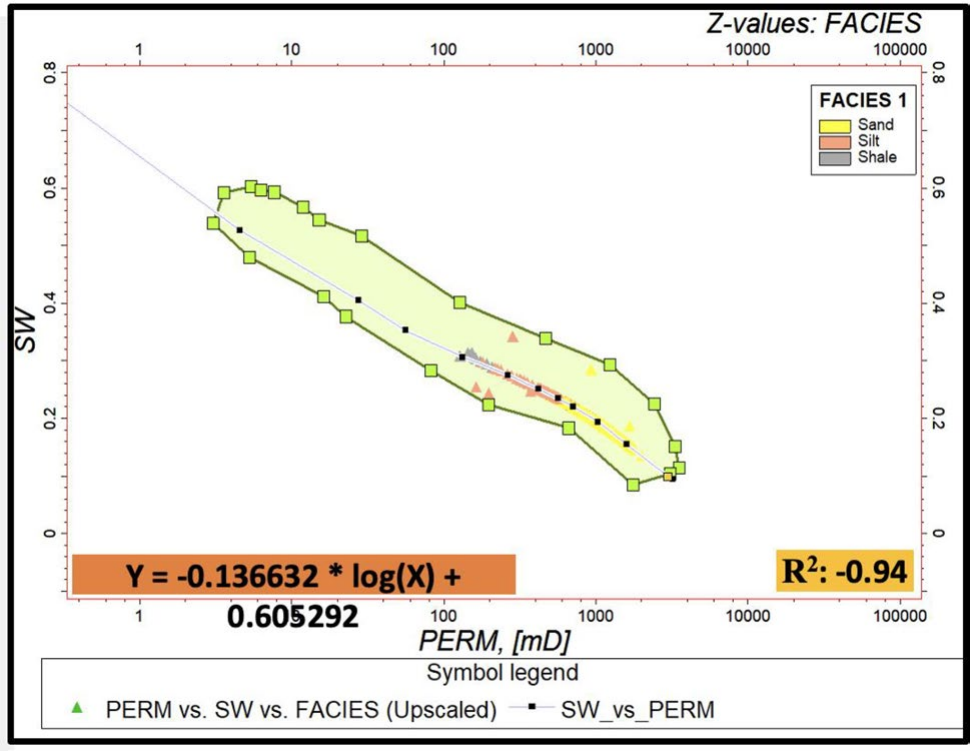
The Crossplot interface for generating water saturation model.

The initial step involves establishing correlations between these logs and core data (from previous study) to establish empirical relationships^22,23^. It is crucial to calibrate seismic attributes with well log data to establish a connection between seismic responses and petrophysical properties. For the modeling of effective porosity, the integration of neutron and density logs with seismic-derived acoustic impedance values is utilized. Permeability estimation involves incorporating porosity data alongside other well logs, such as resistivity and sonic logs, into established permeability models. Water saturation models are created by integrating the water saturation log with the Permeability log Crossplots coloured with Facies with a correlation coefficient of 94%. The resulting function was used to generate a water saturation model from the permeability model (Fig. 4). The iterative refinement and validation of these models against facies enhance their reliability, providing a robust framework for reservoir characterization^24^.

#### Depositional context

Sediments originating from diverse depositional environments can be broadly categorized into continental, shoreline/transitional, and marine deposits. Each depositional system displays distinctive architectural features characterized by specific sedimentological patterns, influenced by sediment transport processes and subsequent deposition within various environmental settings. The shapes, sizes, orientation, continuity, and other attributes of the reservoir are outcomes of sediment transport dynamics, the nature of the depositional environment, basin configuration, tectonic activity, variations in eustatic sea levels, and climatic conditions^4,24^.

Tackling the discerning capabilities of well logs, the task at hand entails the identification and characterization of subsurface lithology and the inherent reservoir fluid typology^2^. The intricate patterns manifested within well logs served as the springboard for establishing the stratigraphic framework and deciphering the trends associated with the lateral continuity prevalent within the field. Intriguingly, the GR (Gamma-ray) curve pattern emerged as an instrument for expounding the depositional environments^25^. Within this context, distinctive curve shapes unfold the Cylindrical shape, marked by minimal GR values, distinct sharp boundaries, and a notable absence of trends; the Bell-shaped, epitomizing an upward augmentation in GR values; and finally the Funnel-shaped, characterized by an ascending decrease in GR values coupled with a coarsening upward succession.

### Results and discussion

#### Reservoir lithology

**Figure 5:**
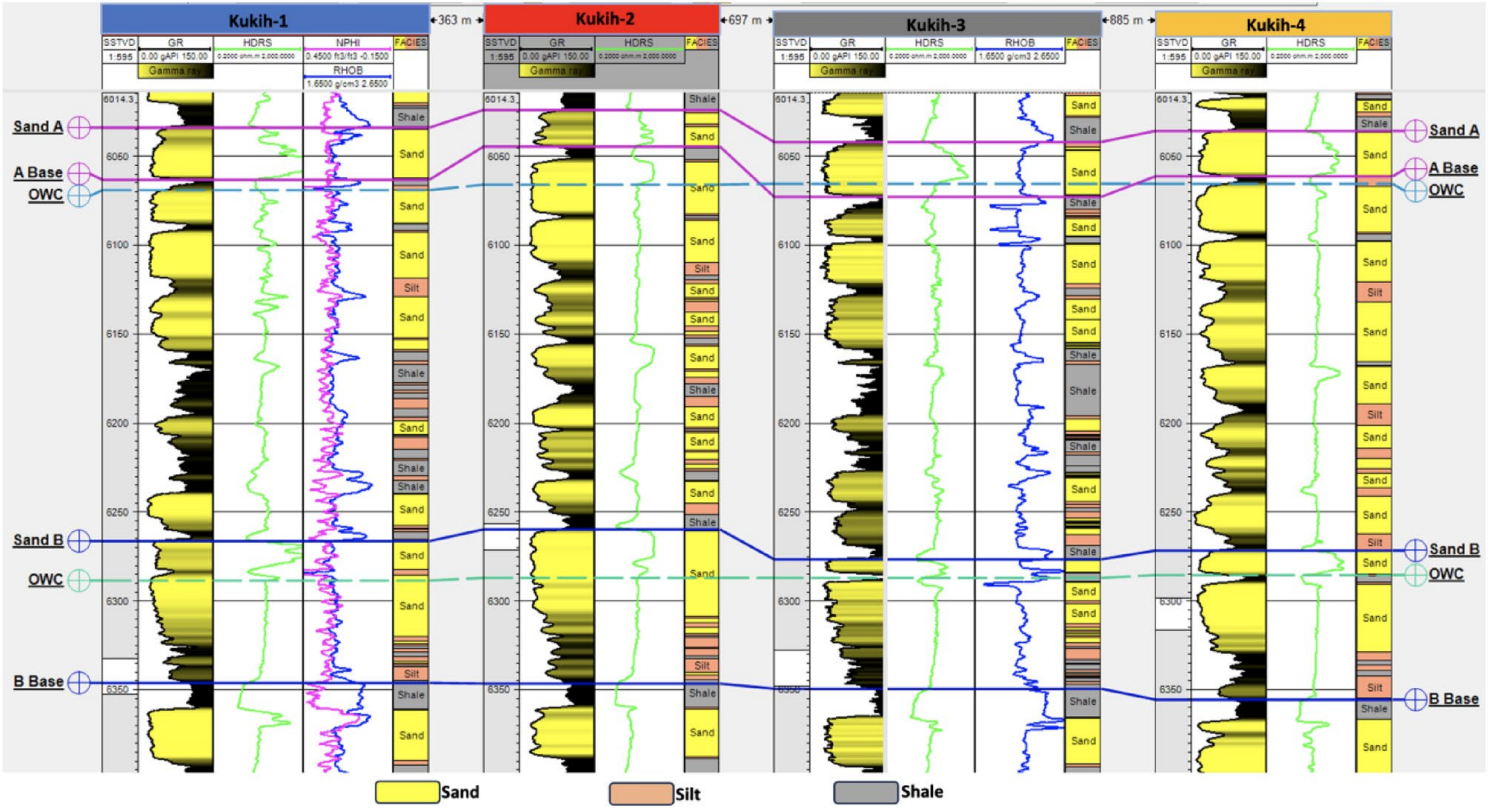
Identified lithologies (denoted as facies) of mainly sand, shale, and silt intercalations on reservoirs sands A and B across the four wells. It also shows the top and bottom of the reservoir sands marked ‘Sand A’ and ‘A Base’ and ‘Sand B and B Base’ Note: OWC is Oil–Water-Contact.

Across the range of wells (K-1, K-2, K-3, and K-4), a discernible dual lithological composition emerged, delineated from uppermost to lowermost layers. The dominant lithologies observed are sand and shale, intercalated throughout the wells, with a minor presence of silt observed within specific segments (Fig. 5). Remarkably, three consistent horizons characterized by sand deposits were distinctly identified and cartographically represented within all four wells (labelled as A, B, and C). These horizons, encapsulated by shale encasements, are recognized as reservoir source rocks. The lithological fabric echoes the geologic narrative of the Oligocene–Miocene ages within the Agbada Formation of the Niger Delta, aligning with the findings of ^26^.

#### Reservoir gross thickness

Probing the dimensions of the reservoir's gross thickness unearthed variations across the wells, as discerned from Fig. 5 and Table 3. Notably, K-2 stands out with an elevated average gross thickness of 24.75 m. In contrast, sands A and C exhibit more modest thicknesses of 7.25 m each. Furthermore, a salient trait emerges in the form of uniform lateral thickness, in consonance with the tenets expounded by^27^.

**Table 3:** Result of petrophysical evaluation of Kukih Field with four hydrocarbon wells and three sands each.

Wells	Sand	Top TVDSS (m)	Base MD (m)	Gross Thickness (m)	Vshale (%)	Vshale (m)	Shale Density (g/cc)	Net Sand (m)	N/G (%)	Total Porosity (%)	Eff. Porosity (%)	Water Saturation (%)	Permeability (mD)	HC Saturation (%)	Fluid Type	S_wirr_
K-1	A	1840	1849	9	17	1.53	33	7.47	83	40	30	31	1308.3	69	Oil + Water	0.63
	B	1910	1934	24	44	10.56	47	13.44	56	29	15	39	1518.9	61	Oil + Water	0.60
	C	2046	2056	10	24	2.4	35	7.60	76	40	25	33	726.8	67	Oil + Water	0.64
K-2	A	1840	1843	3	14	0.42	26	2.58	86	35	31	19	1105.8	81	Oil + Water	0.63
	B	1908	1934	26	31	8.06	52	17.94	69	10	5	18	1283.5	82	Oil + Water	0.56
	C	2047	2053	6	27	1.62	97	4.38	73	35	22	26	612.7	74	Oil + Water	0.64
K-3	A	1841	1851	10	9	0.9	29	9.10	91	32	28	21	847.3	79	Oil + Water	0.64
	B	1913	1937	24	10	2.4	97	21.60	90	30	12	37	302	63	Oil + Water	0.64
	C	2051	2058	7	29	2.03	35	4.97	71	30	15	27	304.5	73	Oil + Water	0.64
K-4	A	1840	1847	7	35	2.45		4.55	65	-	-	-	-	-	-	-
	B	1912	1937	25	42	10.5		14.50	58	-	-	-	-	-	-	-
	C	2049	2055	6	30	1.8		4.20	70	-	-	-	-	-	-	-

NB: The dash ( −) lines imply the absence of well data.

#### Volume of shale (${{\varvec{V}}}_{{\varvec{s}}{\varvec{h}}}$)

**Figure 6:**
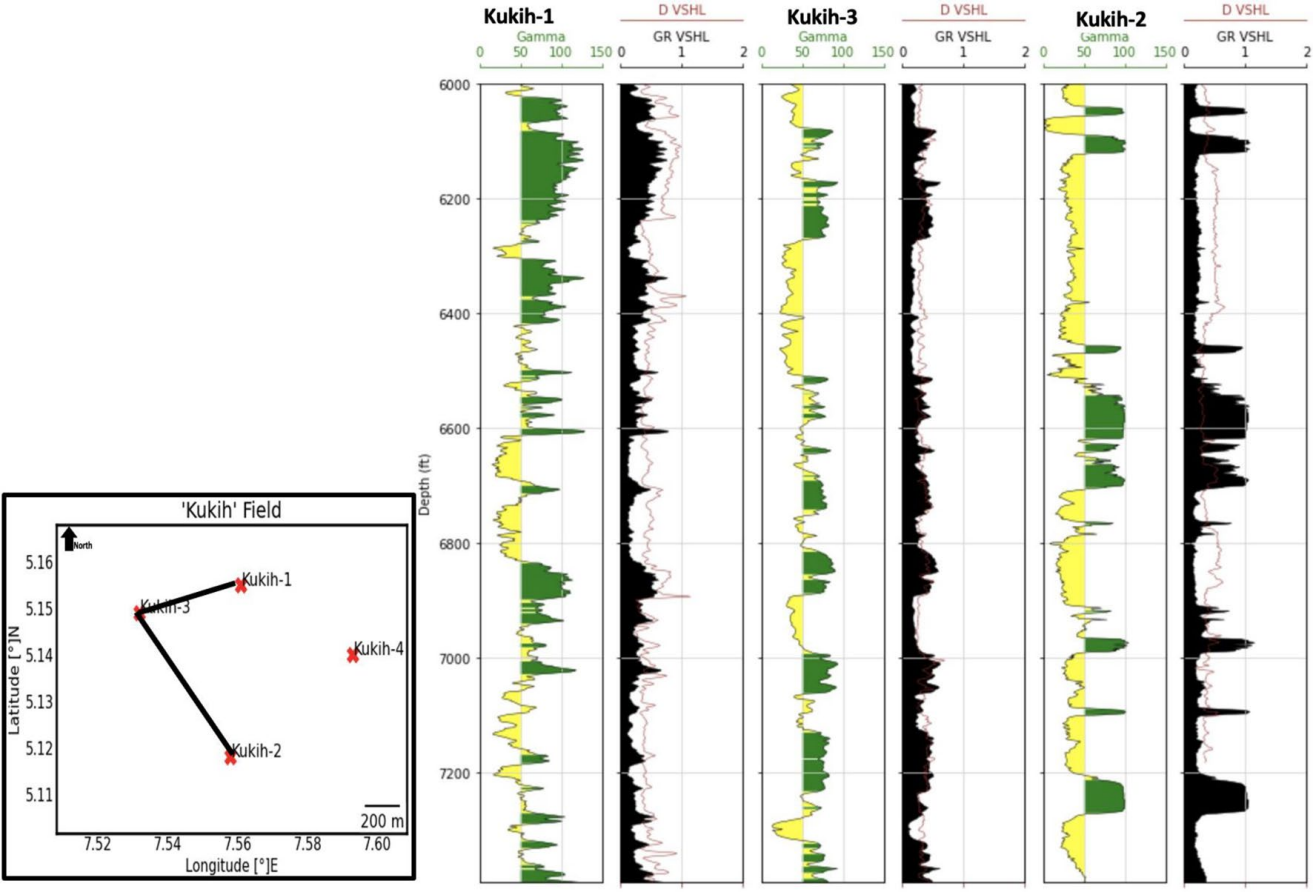
Distribution of shale across Kukih-1, Kukih-2, and Kukih-3 with lithology from Gamma-ray log. NB: D_VSHL is the Density of shale. GR_VSHL is the shale volume. (GR cut-off = 70 API, Vsh cut-off = 0.35 after ^32^). Kukih-4 is not included due to the unavailability of relevant logs.

As a pivotal parameter, the volume of shale (${V}_{sh}$) charts the extent of shale presence within the reservoir. The field-wide distribution of ${V}_{sh}$ indicates conspicuous tendencies of low shale volume, an observation substantiated by the data presented in Tables 3 and 4. This finding is of paramount significance, as diminished shale presence augurs well for the flow of hydrocarbons, thereby enhancing reservoir quality. Among the sands, sand A stands distinguished with the lowest ${V}_{sh}$, whereas sands B and C reveal higher volumes, indicative of a relatively lower impediment to hydrocarbon movement. It is noteworthy that sand A's hydrocarbon-bearing potential is bolstered by its superior quality, underscored by a notably low shale density of 29.3%. This observation gains further clarity as we juxtapose litho-saturation of ${V}_{sh}$ and shale density with lithological attributes, as illustrated by the Gamma-ray log. This visualization underscores the lateral consistency of ${V}_{sh}$ and shale density across the reservoirs, characterized by a linear relationship among Gamma-ray readings, ${V}_{sh}$, and shale density (Fig. 6).

**Table 4:** Reservoir averages of petrophysical evaluation of Kukih Field.

Sand	Gross Thickness (m)	Vshale (%)	Shale Density (g/cc)	Net Sand (m)	N/G (%)	Total Porosity (%)	Eff. Porosity (%)	Porosity Classification	Water Saturation (%)	Permeability (mD)	Permeability Classification	HC Saturation (%)	S_wirr_
A	7.25	13.33	29.33	6.38	86.67	35.67	29.67	Excellent	23.67	1087.13	Excellent	76.33	0.63
B	24.75	28.33	65.33	17.66	71.67	23.00	10.67	Fair	31.33	1034.80	Excellent	68.67	0.60
C	7.25	26.67	55.67	5.65	73.33	35.00	20.67	Very Good	28.67	548.00	Excellent	71.33	0.64

#### Net-to-gross ratio (N/G)

The pivotal metric of net-to-gross ratio (N/G) unveils the proportion of reservoir exploitable in relation to the total reservoir volume. High N/G percentages correspond to reservoirs of superior quality. As revealed by Table 3, well K-1 has an average N/G ratio of 71.6%, followed by K-2 with an average of 76%, and K-3 leading the spectrum with an average of 84%. A significant differentiation within sands becomes apparent, with sand B exhibiting the lowest N/G ratio of 71.67%, while sands A and C manifest ratios of 86.67% and 73.33%, respectively (Table 4). This collective insight reaffirms the predominantly pristine sand composition characterizing the reservoir rocks, echoing the insights posited by^28^.

Geological phenomena like compaction, cementation, clay presence, authigenic mineral growth, and early diagenesis can lead to rocks characterized by remarkably low porosity levels^29,30^. These reduced porosity values exert a significant influence on fluid storage, permeability characteristics, reservoir quality, production methodologies, and the overall geological understanding in the context of hydrocarbon exploration and production^31^. Such low-porosity formations can impede fluid movement, alter reservoir behaviour, and necessitate specialized extraction techniques, underscoring the critical role of porosity assessment in determining the viability and success of hydrocarbon reservoirs.

### Net thickness analysis

**Figure 7:**
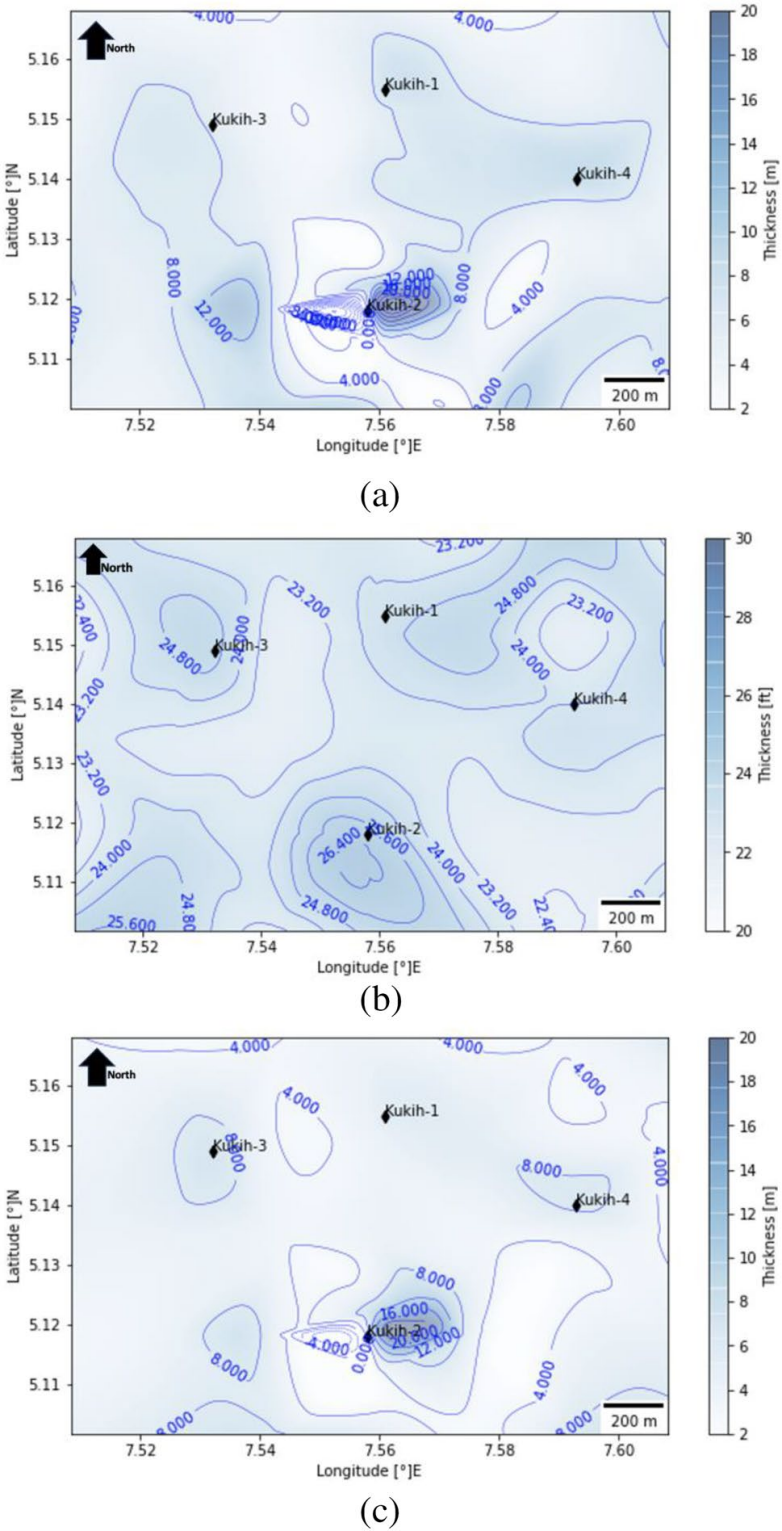
(**a**) Net thickness isopach contour map of Kukih Field (sand A). (**b**) Net thickness isopach contour map of Kukih Field (sand B). (**c**) Net thickness isopach contour map of Kukih Field (sand C). Self-developed with Python [v3.8.8] Programming Language from the Jupyter Notebook [v6.3.0].

Upon the exclusion of shale contributions, a perceptive evaluation of net thickness underscores its critical import. Within this context, the average net thickness measurements reveal important distinctions: sand A exhibits an average of 6.38 m, while sand B boasts a notably elevated 17.66 m, and sand C attains an average of 5.65 m. These insights lend themselves to a deeper comprehension of the reservoir's structural attributes, affording valuable insights into hydrocarbon-bearing strata. By extrapolating the findings to the isopach map of the Formation (Fig. 7), an intricate complexity of net-to-gross values unfurls across the field. While the entirety of the field demonstrates commendable net-to-gross ratios, a discernible elevation in these ratios is discerned in proximity to well K-2. This spatial tendency resonates with the disposition of the well sites. In scrutinizing sand A (Fig. 7a), a noteworthy trend emerges, K-1 and K-4 exhibit high values, showcasing a progressive rise following the slope from K-2 towards the northeastern direction. Notably, K-3, characterized as a deviated well, offers an intriguing dynamic. While presenting considerable thickness in sand B (Fig. 7b), it registers relatively lower net thickness values in both sand A and sand C (Fig. 7c) (as evident from the well logs in Fig. 5). Clearly, the combination of sand qualities and net-to-gross ratios augurs well for future drilling activities, affirming the overall suitability of the reservoir for such endeavours.

### Lithology and porosity from neutron-density (N-D) cross-plot

The reservoir domain of well K-1 is comprehensively covered by the analytical exploration, which includes the establishment of cross plots from the Neutron and Density logs. These logs are used for lithological characterization due to the well-established lithological uniformity within the Agbada Formation and the absence of gas zones. The Neutron-Density cross plot, shown in Fig. 5, serves as the technique for effectively delineating the lithological composition. To correlate lithological ranges, an overlaid cross-plot chart emphasizes the lithological diversity to be within the sandstone domain, with a maximum matrix density not exceeding 2.65 g/cc^33^. This maximum matrix density serves as a definitive indicator of the reservoir's lithological essence. The visual representation of these cross plots highlights the porosity aspect, where a bulk density less than 2.2 g/cc corresponds to a porosity greater than 25%. This information is crucial in providing a more precise and refined porosity evaluation compared to the conventional density log^34^.

## Results of petrophysical analysis

### Oil–water-contact (OWC)

The delineation of Oil–Water-Contacts (OWC) across the field is comprehensively illustrated in Fig. 5. Particularly, sands A, B, and C demarcate distinct OWC levels positioned at depths of 2002 m, 2075 m, and 2224 m, respectively. In this pursuit, resistivity logs were leveraged to delineate hydrocarbon contacts. However, the limited resistivity contrast posed challenges in mapping hydrocarbon-bearing formations within sandstone reservoirs through conventional logs. This challenge mirrors the observations of^35^, who underscored the qualitative nature of identifying low resistivity zones within older clastic fields, owing to the dearth of advanced logging techniques. But, according to^36–39^, clastic and limestone reservoirs, in contrast to their carbonate counterparts, present intricacies in terms of thickness, influenced by factors spanning porosity, mineralogy, fluid type, and pore geometry. These gradations could potentially engender complications in rendering qualitative estimations of reservoir attributes.

### Porosity analysis

**Figure 8:**
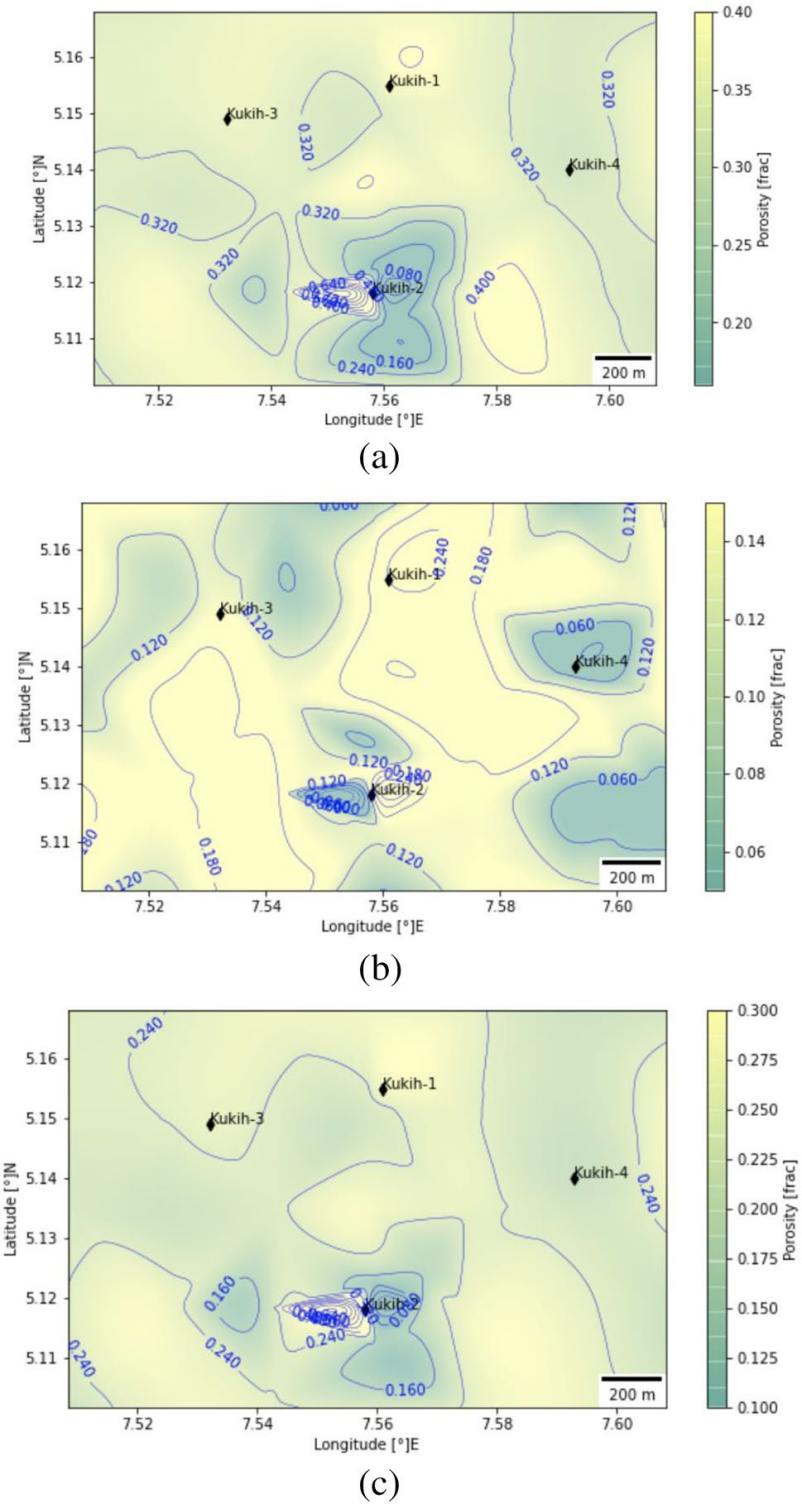
(**a**) Average porosity contour map at reservoir sand A (1840 m–1851 m) for the Kukih Field. (**b**) Average porosity contour map at reservoir sand B (1910 m–1937 m) for the Kukih Field. (**c**) Average porosity contour map at reservoir sand C (2046 m–2055 m) for the Kukih Field. Self-developed with Python [v3.8.8] Programming Language from the Jupyter Notebook [v6.3.0].

The comprehensive analysis of porosity unfolds through the presentation of both total and effective porosity measurements, encapsulated within Table 3, with respective averages encapsulated within Table 4. Across sands A, B, and C, the average total porosity stands at 35.67% and effective porosity at 29.67%. Meanwhile, sand B registers an average total porosity of 23% and an effective porosity of 10.67%, while sand C showcases an average total porosity of 35% and an effective porosity of 20.67%. Drawing insights from^16,40^, we ascertain that sand A exhibits excellent porosity, whereas sand B's porosity quality is classified as fair. In the case of K-2, sand A and C showcase excellent porosity, while sand B's porosity profile veers toward very poor, verging on negligible. Turning attention to K-3, sand A presents excellent porosity, while sand B and C adopt the fair classification (Table 3), concurring with the insights derived from the Neutron-Density porosity cross-plot. The holistic perspective is further accentuated through the presentation of the average effective porosity contour map (Fig. 8a-c), seamlessly capturing the lateral variation in porosity across sands A to C. Intriguingly, sand A exhibits excellent porosity across K-1, K-2, and K-3, with K-1 registering the highest magnitude. A coherent spatial trend emerges within the central, northern, and western segments, collectively spotlighting high porosity values, that are indicative of a porosity entity around K-1 and K-3. Meanwhile, sand B portrays negligible porosity levels at K-2, with K-4's porosity status remaining undetermined. The salient observation is the anticipated basin-wide decline in porosity with increasing depth, evidenced by the porosity reduction between sand A and B within K-1 (30% to 15%), K-2 (31% to 5%), and K-3 (28% to 13%). This corroborates the findings articulated by^41,42^. Conversely, sand C exhibits heightened porosity at K-1 and K-2 compared to sand B. This divergence could be attributed to proximal over-pressured zones, engendering deviations from the anticipated reduction trend. The litho-saturation integration of effective and total porosity with lithology harnessed through the Gamma-ray log, underscores their seamless continuity across the reservoirs. Notably, the porosity distribution exhibits an increasing potential as one progresses from south to north within sand A.

### Formation water resistivity (${{\varvec{R}}}_{{\varvec{w}}}$) determination

**Figure 9:**
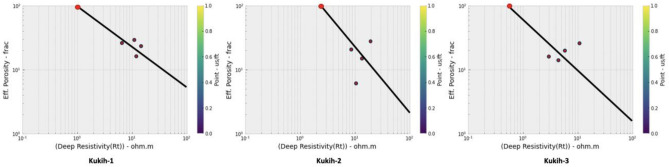
Estimation of Formation Water Resistivity (${R}_{w}$) for Kukih 1–3 using Pickett (linear) plot. *Note* The red dot on the black line represents the value of ${R}_{w}$.

The pivotal task of establishing the Formation Water Resistivity (${R}_{w}$) was undertaken through a dual-pronged approach, combining the insights garnered from the Pickett plot and Archie’s equation, as illustrated in Fig. 9. The ${R}_{w}$ values manifest a discernible span, oscillating between 1.0 and 1.5 Ω-m for K-1, while for K-2, the range spans from 1.1 to 3.0 Ω-m. The trajectory of the gradient line intersects the true resistivity (${R}_{t}$) values, with the Formation’s water resistivity (${R}_{w}$) prominently marked by the red dot, serving as a beacon of ${R}_{w}$'s characterization^35^. Evidently, ${R}_{w}$ registers as 1.1 Ω-m for K-1, 2.5 Ω-m for K-2, and 0.6 Ω-m for K-3.

The resulting ${R}_{w}$ values, spanning an observable range, hold significant implications for reservoir assessment. In the context of K-1, the low ${R}_{w}$ value underscores the validation of hydrocarbon-filled pore spaces. Similarly, in K-2, the range of 1.1 to 3.0 Ω-m highlights the diverse resistivity conditions present. The gradient line's intersection with true resistivity values, signified by the red dot, pinpoints the specific values of ${R}_{w}$, and provides an indispensable reference for reservoir analysis. The distinct ${R}_{w}$ values across K-1, K-2, and K-3 showcase the reservoir's intricate hydrogeological variations and unveil the implication of the Formation water resistivity within this reservoir section. Conversely, the differentiation between observed water resistivities highlights the importance of localized ${R}_{w}$ characterization.

### Fluid saturation assessment (${{\varvec{S}}}_{{\varvec{h}}}$, ${{\varvec{S}}}_{{\varvec{w}}}$, and ${{\varvec{S}}}_{{\varvec{w}}{\varvec{i}}{\varvec{r}}{\varvec{r}}}$)

**Figure 10:**
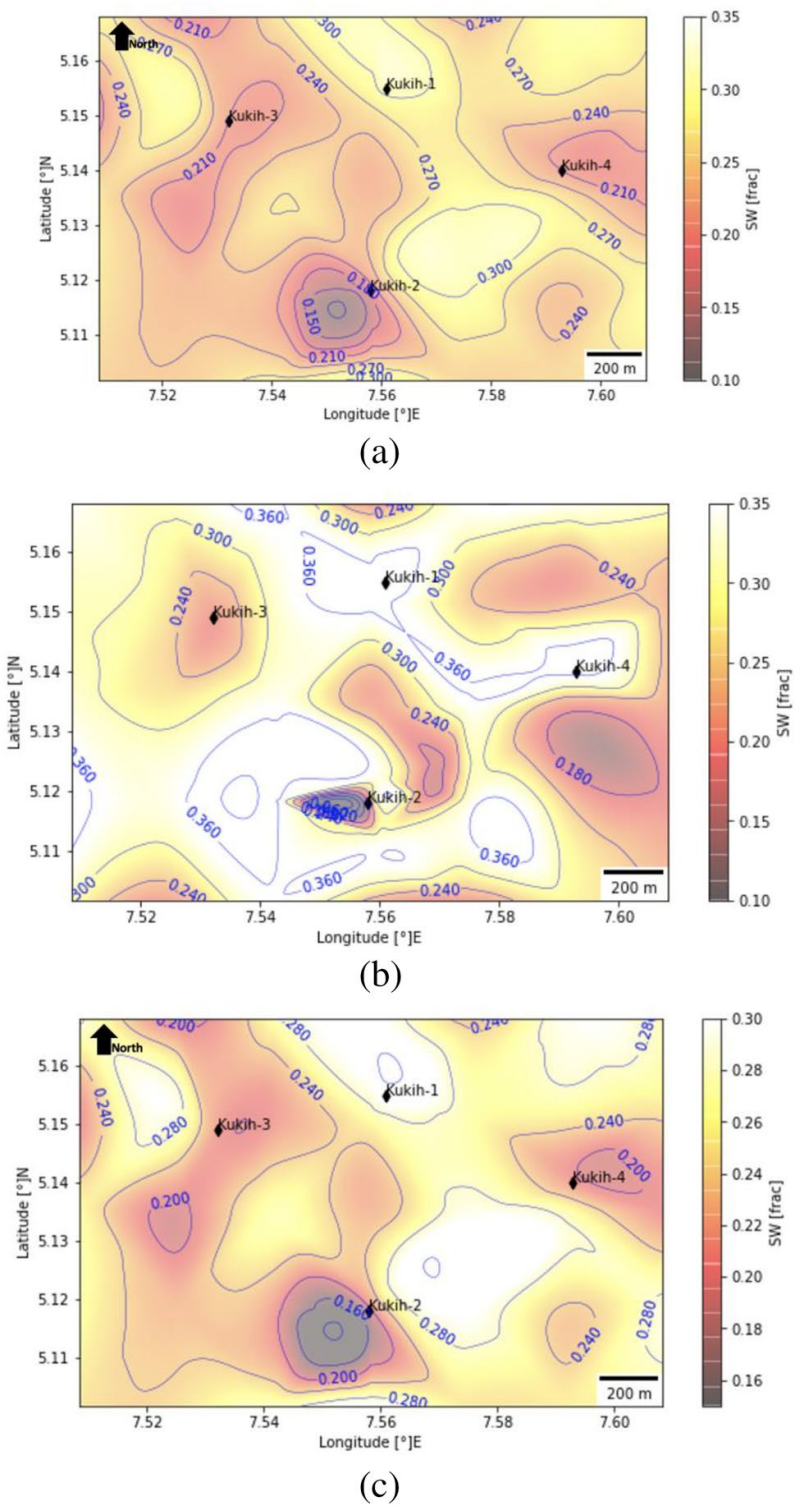
(**a**) Average water saturation contour map at reservoir sand A (1840 m – 1851 m) for the Kukih Field. (**b**) Average water saturation contour map at reservoir sand B (1910 m – 1937 m) for the Kukih Field. (**c**) Average water saturation contour map at reservoir sand C (2046 m – 2055 m) for the Kukih Field. Self-developed with Python [v3.8.8] Programming Language from the Jupyter Notebook [v6.3.0]

Within the ambit of fluid saturation analysis, the examination delves into the intricate interplay of Shale Saturation (${S}_{h}$), Water Saturation (${S}_{w}$), and Irreducible Water Saturation (${S}_{wirr}$), affording a holistic perspective of the reservoir dynamics. Focusing on the water saturation profile of the sand A reservoir, discernible trends emerge, painting a comprehensive spatial narrative. The northern region, particularly around K-1, notably exhibits an elevated water saturation gradient, diverging away from the reservoir's core. In a contrasting vein, K-3 presents a distinct characteristic, markedly lower water saturation levels, an attribute poised to amplify its hydrocarbon saturation. Meanwhile, K-2 emerges as the epicentre of heightened water saturation, extending from the southern flank to the central north, with an anomalous sharply low ${S}_{w}$ to the lower south, an observation vividly materialized within Fig. 10a. In the realm of sand B, intriguing dynamics unravel. K-1 commands the peak of water saturation, while K-2 witnesses a perceptibly diminished ${S}_{w}$. Notably, hydrocarbon flow assumes a transitory trajectory, commencing from K-2, the pinnacle of hydrocarbon saturation, and cascading westward towards K-3. Interestingly, when we progress toward the northern boundary of the study area, we observe a notable decrease in hydrocarbon saturation. This phenomenon is meticulously illustrated in Fig. 10b and consistently observed in Fig. 10c. Additionally, there is a distinct pattern of relatively lower water saturation (${S}_{w}$) throughout the field, with a more pronounced contrast evident at location K-4. The elucidation of the irreducible water saturation (${S}_{wirr}$) unfolds as a cardinal determinant in the calculus of reservoir productivity. ${S}_{wirr}$'s quantification hinges upon the reservoir sands' permeability and effective porosity, elaborately shaping the maximum producible water content concomitant with oil extraction. Remarkably, the ${S}_{wirr}$ parameter assumes paramount significance in operational economics, and a higher ${S}_{wirr}$ imparts increased production costs, thereby underscoring the economic implications associated with field exploitation.

### Permeability analysis

A comprehensive assessment of permeability, meticulously detailed within Table 4, showcases insights that align with the characterization posited by^17^. Evidently, the permeability manifests a high-grade quality, synergistically conducive to fluid mobility. The permeabilities average, distinctly assigned to sand A, B, and C, register at 1087 mD, 1035 mD, and 548 mD, respectively. The overarching trend underscores a propensity towards permeability reduction with increasing depth, an observation that echoes established geological tenets. Notably, the permeability contour map harmoniously mirrors the observed trajectory of water saturation flow, substantiating their proportionate relationship.

### Seismic interpretation

**Figure 11:**
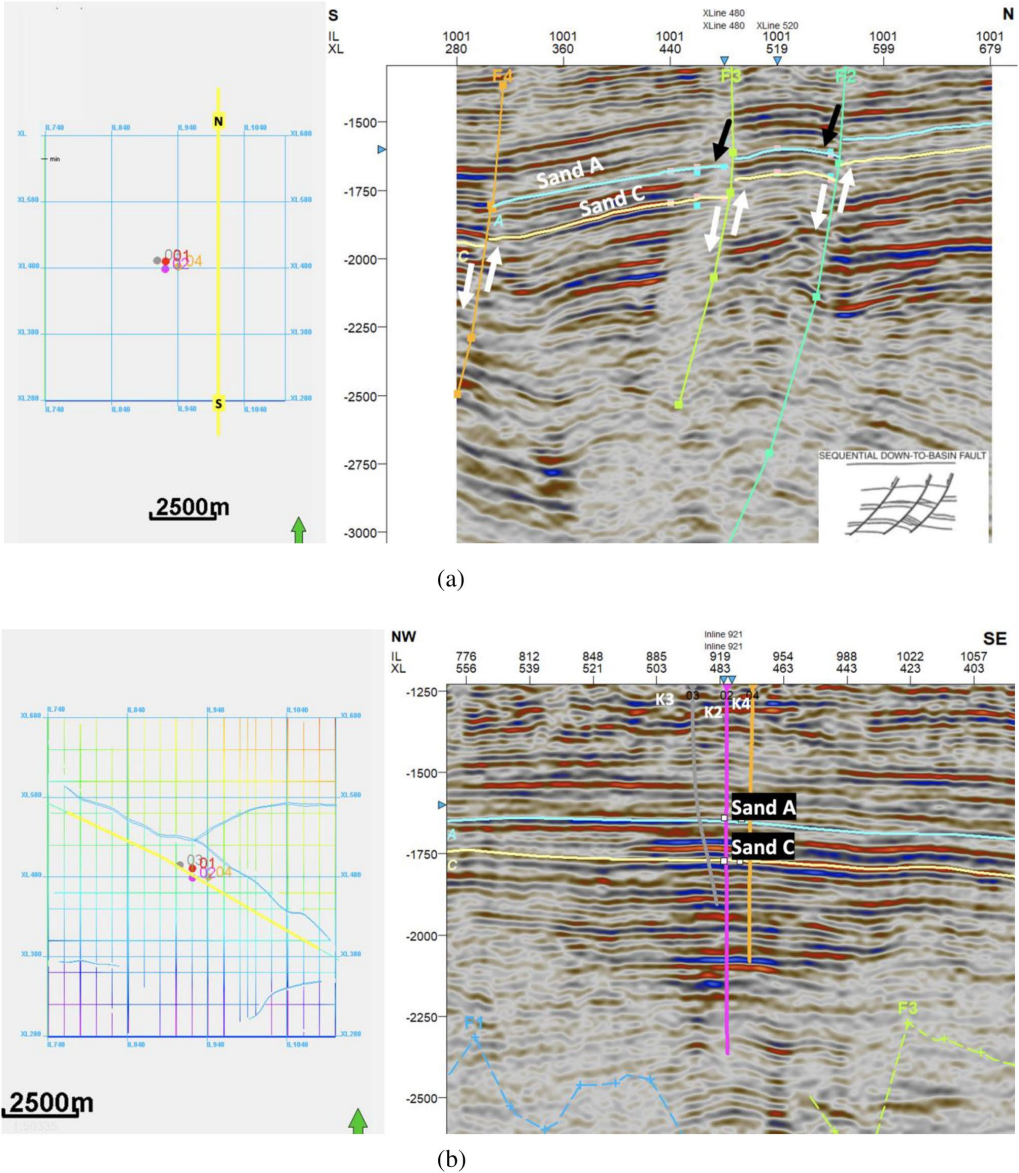
(**a**) Horizon and Fault Interpretation on Inline 1001. A and C are mapped horizons. F2, F3 and F4 are sequential-down-to-basin Growth faults in the time domain, with the direction (N-S) indicated on the location mapped. Dip directions are indicated with white arrows. Sequential-down-to-basin faults are indicated with black arrows. (**b**) Interpreted horizon interval across Growth Fault on Strike Line (shown as yellow line on location map). F1 and F3 are faults. K2 and K4 are vertical wells. K3 is a deviated well. Sand A and C are mapped horizons.

**Figure 12:**
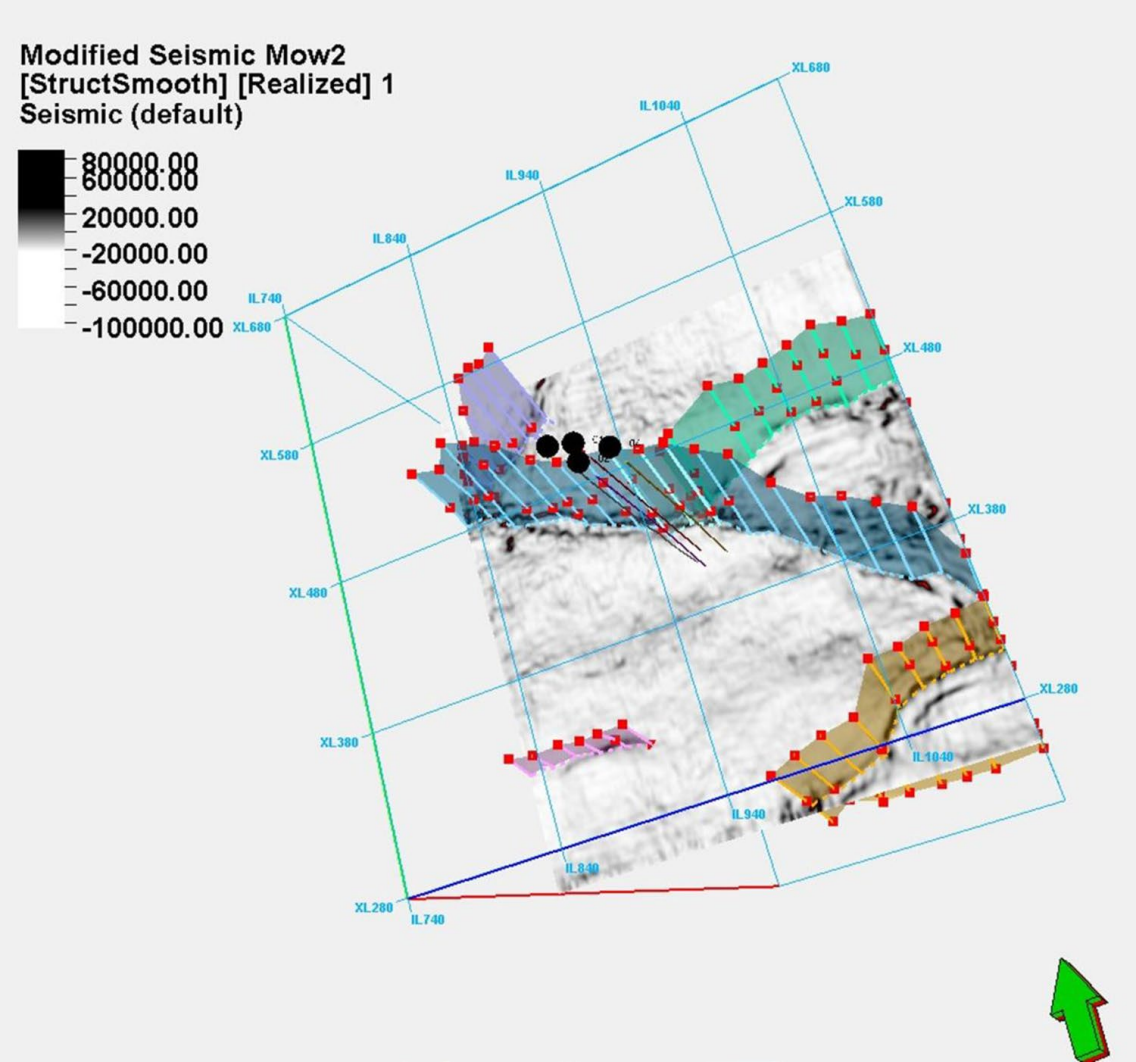
Synthetic and antithetic faults with inserted well logs from the Kukih field in the depth domain. This diagram was generated using Petrel [v2019.1].

The reservoir landscape crystallizes into sharper focus through the cortex of seismic data interpretation. The canvas unfolds a panoramic expanse, vividly encapsulating the potential reservoir reach. Anchoring upon a 3-way fault-assisted closure mechanism, the reservoir's trapping dynamic attains distinct articulation. This intricate orchestration culminates in a straightforward rollover anticline configuration, forged upon the downthrown flank of a prominent Northwest-Southeast trending, sequential-down-to-basin growth fault. In congruence with the anticipated behaviour of growth faults, the seismic analysis delineates the hallmark of sediment thickening, intriguingly distributed across the fault line. This augmentation in sediment accumulation can be ascribed to the syn-depositional attributes inherent to sequential-down-to-basin growth faults^43^. The seismic data scrutiny shows synthetic faults; F2, F3, and F4, etched across horizons A and C on the seismic cross-section (Fig. 11 a and b). This pivotal observation is further buttressed by the documentation of significant structural constituents, epitomized by the prominent synthetic and antithetic faults delineated within Fig. 12. These intricate features manifest a consistent northwest-to-southeast dip, unveiling the cardinal role these geological elements play within the field's structural complexity. The compositional landscape additionally unveils the presence of minor faults, punctuated by minor structures, a significant observation within the broader context of the Kukih field. The depth and intricacy of the 3D seismic analysis furnish a privileged vantage point, affording an unprecedented level of detail regarding the field's structural complexities.

A notable observation emerges; the presence of a major growth fault labeled as F2, traversing the entire expanse in a Northwest-Southeast orientation (Fig. 11a). This prominent fault serves as a support, conferring structural cohesion upon the geological landscape. Complementing this, subsidiary faults, emerge in placement with the central growth fault (Fig. 12). Remarkably, the synthetic faults display an extensional nature, dipping toward the southwestern direction and exhibiting parallel or sub-parallel alignment. This intricate interplay resonates with the Niger Delta's characteristic stratigraphic arrangement, characterized by alternating shale and sand sequences. These structural elements correspond harmoniously with the Niger Delta's unique depositional pattern. Notably, these structures perform a dual role, connecting stratigraphic layers and facilitating the transmission of hydrocarbons. This functional versatility ensures the seamless passage of hydrocarbons from the Akata Formation to the Agbada Formation.

**Figure 13:**
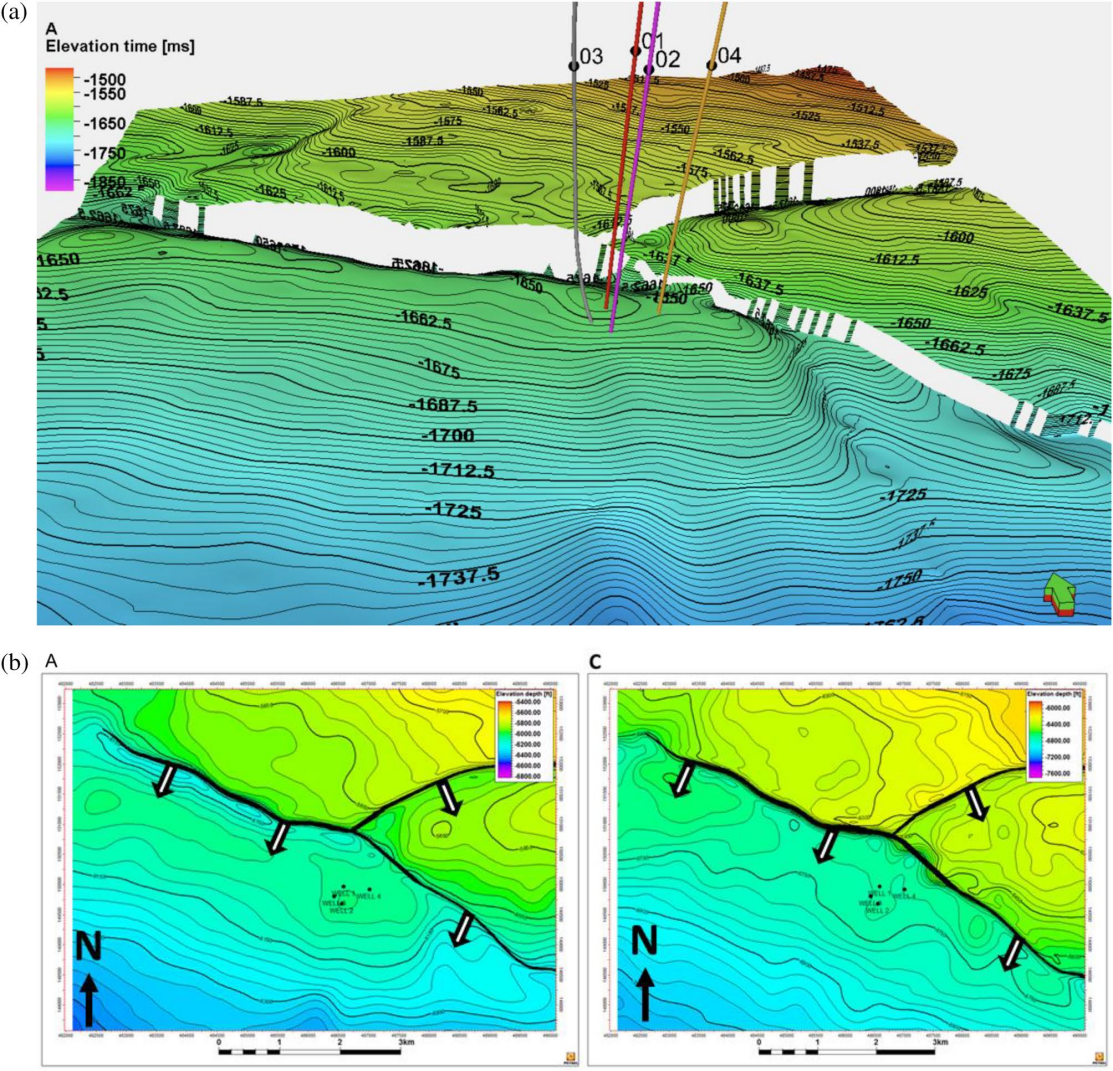
(**a**) Three dimension time structure map of horizon A. (with wells Kukih-1, Kukih-2, Kukih-3, and Kukih-4 denoted as 01, 02, 03 and 04). (**b**) Structural top depth map of the velocity model conversion of horizons A and C (with wells Kukih-1, Kukih-2, Kukih-3, and Kukih-4 denoted as Well 1, Well 2, Well 3 and Well 4) It highlights the fault with the dip direction (black arrows) in a 2D section map.

**Figure 14:**
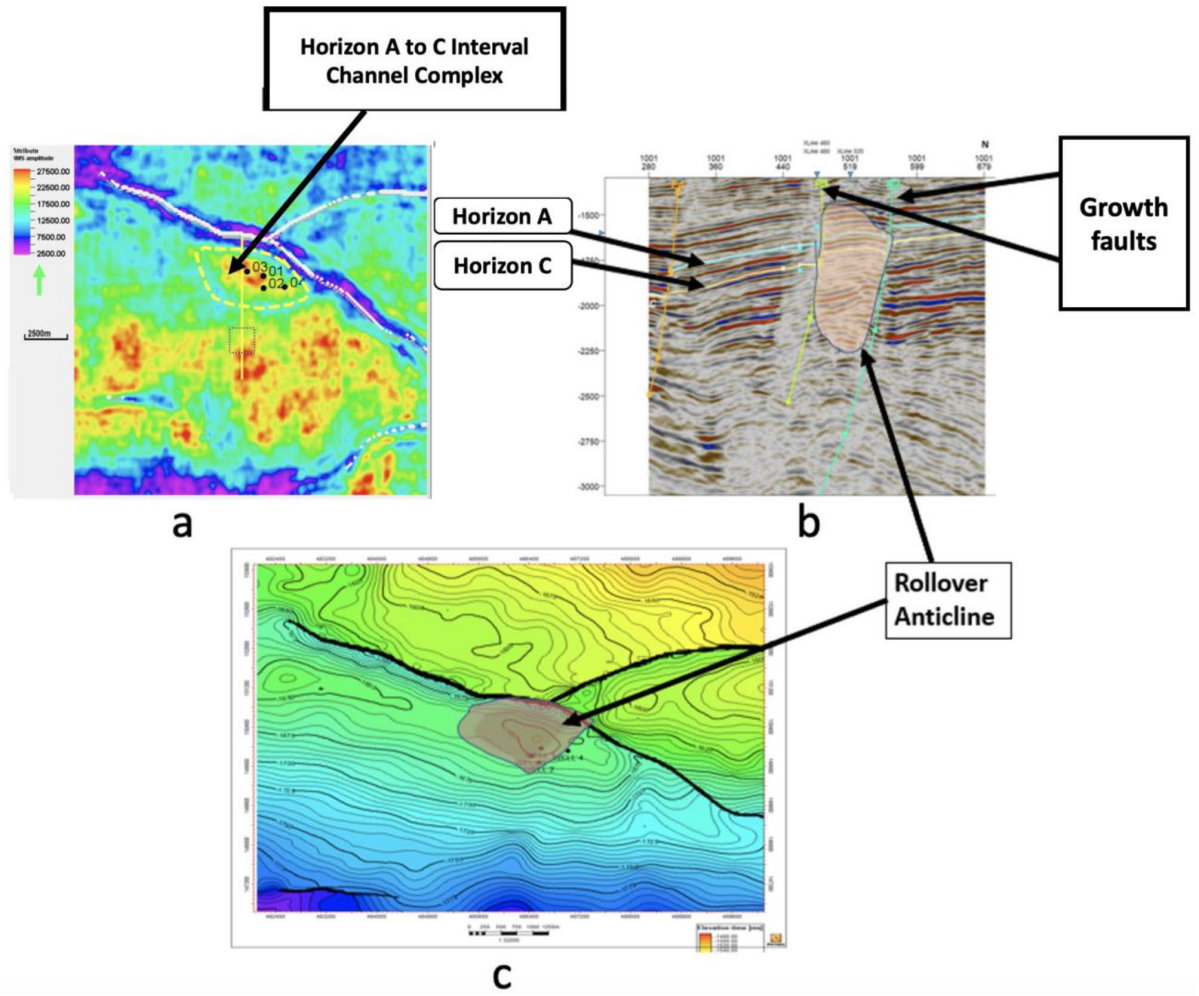
Channelized complex with rollover anticline and the faults acting as hydrocarbon traps. (**a**) Horizon slice map with channelized sand complex. (**b**) Seismic section with growth faults (**c**) Depth map with major faults.

**Figure 15:**
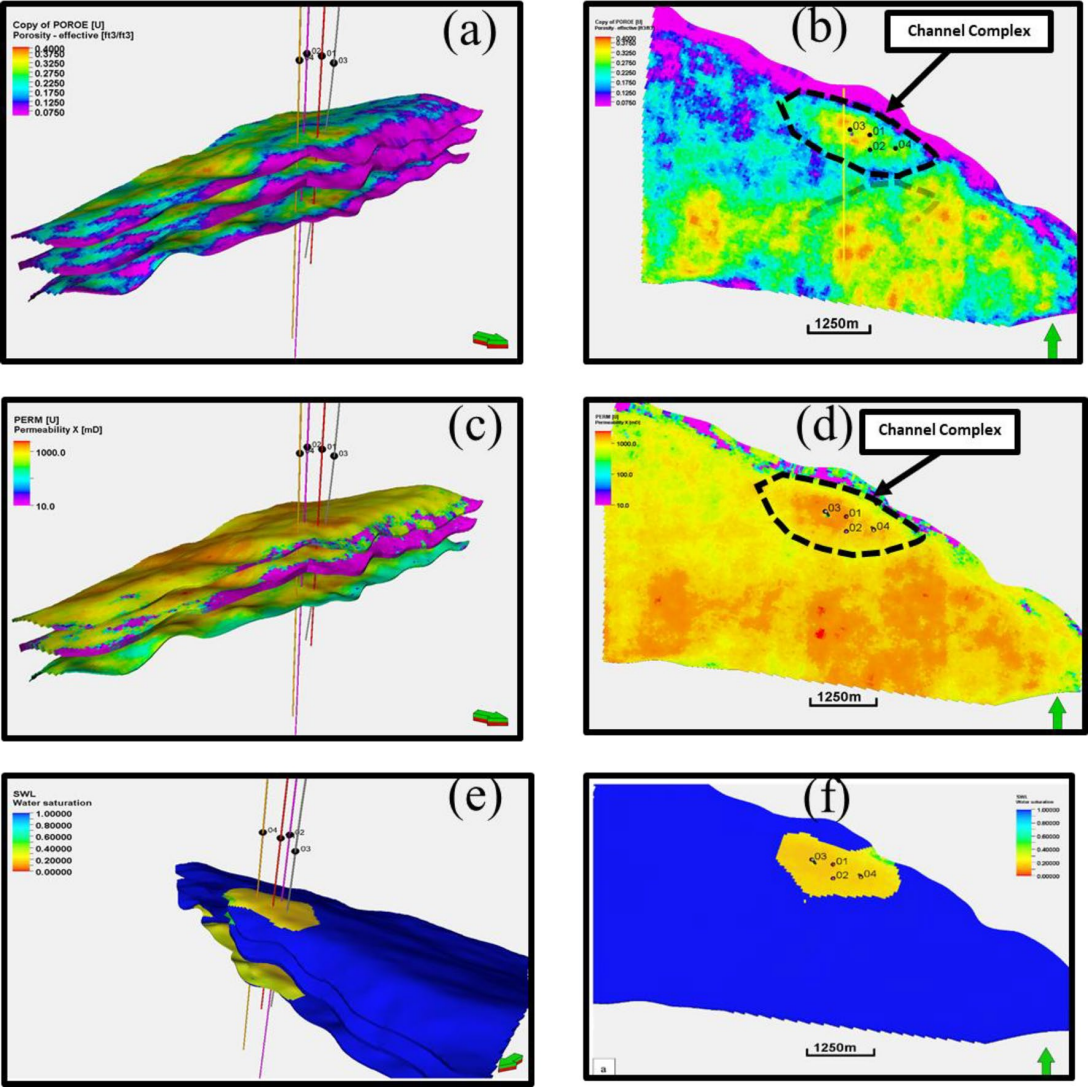
(**a**, **b**) effective porosity model (3D and 2D views), (**c**, **d**) permeability model (3D and 2D views), (**e**, **f**) water saturation model (3D and 2D views generated from the permeability model).

The structural time and depth map, depicted in Fig. 13a, b, is crucial in assessing the alignment between prospects in horizon A and the underlying structure. It scrutinizes the consistency between the prospects' configuration and the prevailing structural features. Structural insights from Fig. 13a unveil a significant outlook, delineating distinct features within the study area. The central expanse unfolds as a structural high, demarcated by the encompassing embrace of a growth fault to the southwest. In counterpoint, the northeastern area reveals a bifurcated landscape, marked by two low intra-reservoir faults, separated by an antithetic fault, coalescing into a complex configuration of three-way fault zones. This intricate amalgamation converges to craft a channelized fault reservoir dynamic (Fig. 14). The strategic placement of the deviated well, K-3, emerges as a tactical manoeuvre necessitated by the reservoir's fault-infused architecture. Its trajectory is seemingly directed to intersect a reservoir structure, an astute response to the intricate reservoir fault geometry. The reservoir fabric, embodied within reservoir A , unveils channelized sands that traverse from northwest to southeast, an articulate assertion to the underlying channel trajectory (Fig. 14a, b. The point of reservoir potential resides within the southwest subsection; a principal reservoir stronghold, whereas the two northeastern subsections epitomize reservoir prospects of distinct promise. A focal point of the petrophysical endeavour lies squarely upon the principal reservoir subsection (Fig. 14c). This pivotal region, marked by low shale volume, high porosity, low water saturation, and robust sand thickness across horizons A and C, attests to its intrinsic reservoir quality. Remarkably, the petrophysical model emerges as a corroborative sentinel and agrees with the well data's properties as shown in Fig. 15. This synthesis underscores the interplay of geological complexities and reservoir properties, revealing a reservoir characterization narrative that resonates with empirical data^38^.

### Facies and depositional context

**Figure 16:**
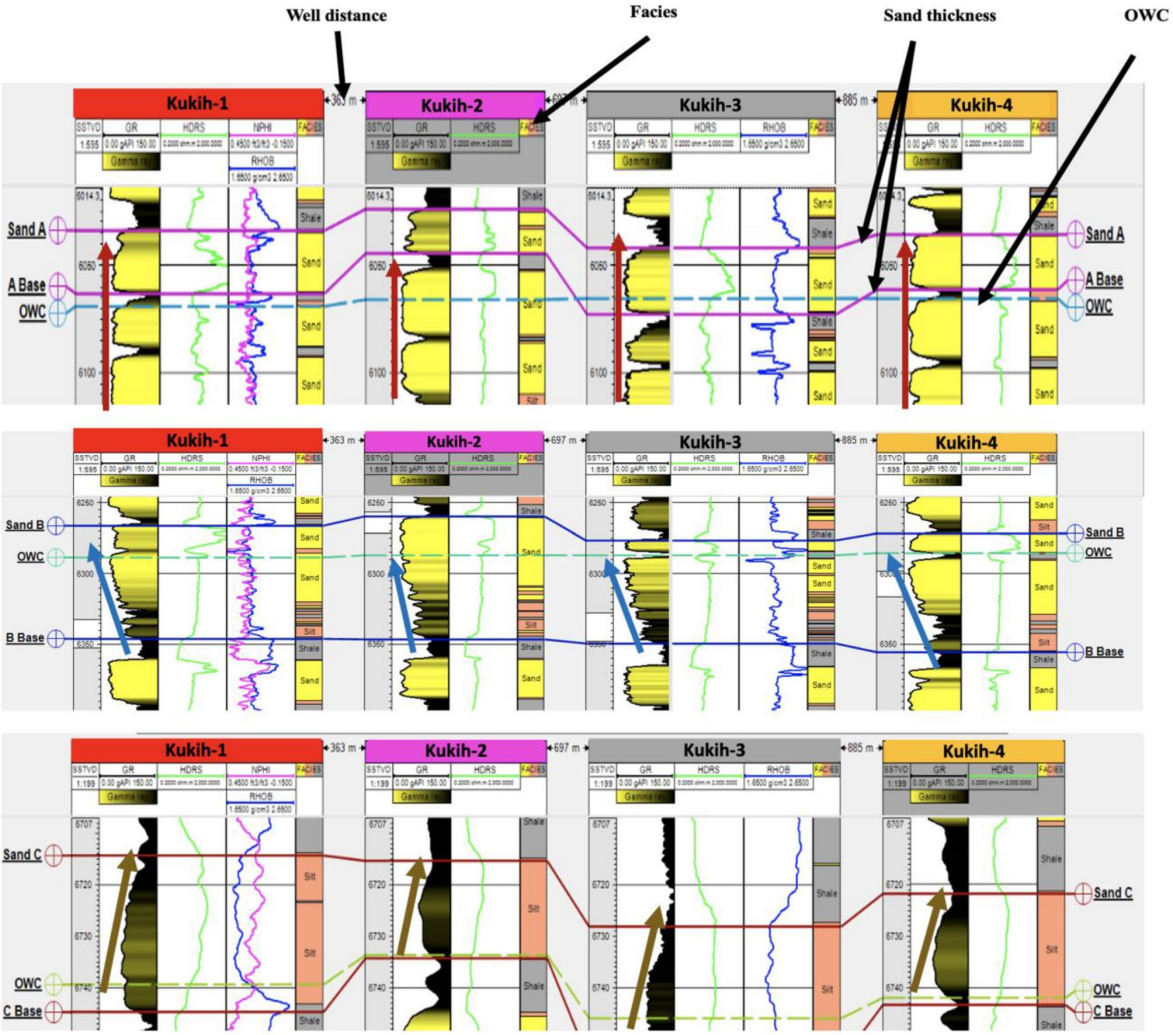
Depositional environment for Sands A, B, and C.

Figure 16 provides a visual indication of the prevailing facies, aptly suited to a channelized environment. At different depths, varying gamma-ray shapes, representing the different environments of deposition were observed. A distinctive assemblage of depositional signatures emerges, as shown in Fig. 16 (Sand A). Evidently, a notable piece of serrated cylinder-shaped successions was unveiled in reservoir sand A, etching a thematic form of continuity. This cylindrical motif, endowed with a thickness spanning 14–19 m across the well cross-sections, gravitates toward depths ranging from 1839 to 1859 m. Reminiscently, this serrated cylinder motif, a characteristic of well logs tracing their lineage that is related to the deep-water sedimentary environment, imparts a resonance with submarine channels and fans. This symbolic motif conveys the cyclical rhythm of sediment deposition intrinsic to these dynamic environments, suggesting a relationship with slope channels or inner fan channels. Of particular significance, the prominence of cylindrical contours suggests the dominance of turbidite sands, a domain fundamentally attached to the upper units of the Niger Delta's Akata Formation, serving as a repository of deepwater reservoir sands^8^.

Figure 16 shows the depositional environment of Sand B, meticulously deciphered from the gamma-ray logs across K-1 to K-4. This manifestation is observed within an average depth span ranging from 1917 to 1938 m, with a thickness between 18 and 24 m, culminating at approximately 21 m. Conspicuously, this arrangement unfolds as a serrated funnel-shaped progression, characterized by a parallel alignment to the shoreline. Upon a vertical view, the fabric of turbidite deposits unveils a distinctive trend, amplifying sand content towards the upper parts of the deposition. This propensity, widely recognized as 'cleaning upwards,' is a hallmark feature resonant within deep marine environments. Noteworthy instances of smaller thicknesses, clustering within 6–10 m, might connote crevasse play, while more expansive extents, surpassing 40 m, suggest the notion of a prograding delta. Within this interpretive point of view, insights gleaned from^44^ converge. These observations underscore aggradation sequences, encompassing sands, shales, and silt constituents, typical of the hinterland basin environment forged through suspension deposition. Though glauconite and mica are notably absent, the assessment's completeness is limited by the paucity of cores, which hinders definitive confirmation. Notably, the nobility of this succession, along with its compositional aggregation, sternly aligns with the contours of a prograding delta, a classification that finds validation within Rider's taxonomy^40^. This deduction encapsulates the intricate interplay of facies and depositional contexts, weaving a narrative anchored in empirical insights and geological understanding.

Similarly, the stage is equipped with the slightly serrated bell motif, a symbol that is prominent within reservoir Fig. 16 (Sand C), observable across wells 1 to 4. While the bell-shaped sequence represents diverse sedimentary environments, from transgression sands to tidal or deep tidal channels, and even fluvial or deltaic channels, these motifs transcend customary boundaries. The geology, fortified by its nonalignment with carbonaceous debris, cast aside the areas of fluvial or tidal channels. Likewise, glauconite and shell debris, typically woven into the complexity of tidal channels, contradicts such affiliation. Weber's sedimentation cycle model surfaces, showing a content formed by the erosion of underlying sand formations and underscored by the distinct unconformity that accompanies the subject of fossiliferous transgressive sea sands^45^. This motif diminishes the likelihood of a continental depositional environment, evident in the prominently elevated gamma-ray values indicative of heightened radioactivity originating from organic matter present in marine shales and sediments. The observed cyclic pattern in the gamma-ray log is attributed to variations in sediment types associated with alternating sea levels and distinct depositional conditions. Figure 16 (Sand C) concludes in the tranquil embrace of a transgression marine shelf environment. Table 5 summarises the depositional environment for the sand units (designated as A, B, and C) within the Well motifs, categorizing them as Cylinder, Funnel and Bell^46^.

**Table 5:** Summary of the depositional environment from the well motif in Kukih Field, adapted after ^3^.

Sand unit	Kukih-1	Kukih-2	Kukih-3	Kukih-4
A	Cylinder	Cylinder	Cylinder	Cylinder
B	Funnel	Funnel	Funnel	Funnel
C	Bell	Bell	Bell	Bell

### Recommendation for further studies

Drawing from the integration of seismic interpretation and supportive petrophysical analysis, several high-potential exploration zones warrant further investigation. Especially, improved fluid contact analysis on water saturation model should be performed before further volumetric analysis and exploitation engagements^47,48^ Priority should be given to channelized reservoirs displaying distinctive Cylinder- and Funnel-shaped facies, while structural traps and fault zones aligning with favourable reservoir properties should be thoroughly examined. Areas exhibiting lateral porosity variation, particularly southwestward, present alluring prospects, along with zones characterized by low shale volume and promising porosity–permeability relationships. Additionally, the intersection of reservoir horizons should be scrutinized for elevated hydrocarbon potential. These recommendations synergistically harness seismic insights and reservoir characteristics, guiding strategic exploration endeavours for optimal outcomes.

## Conclusions

The integrated reservoir characterization study conducted on the Kukih Field in the Onshore Niger Delta, Nigeria, has yielded a thorough comprehension of its geological and hydrocarbon potential. By combining well-log data and 3D seismic interpretation, distinct lithological units have been identified, with sand and shale prevailing across multiple wells. Petrophysical assessments, including parameters such as volume of shale (V_sh), net-to-gross ratio (N/G), and net thickness, have revealed the superior hydrocarbon-bearing potential of specific sands, particularly Sand A. This study's innovative approach, integrating petrophysical analyses with seismic data interpretation, has not only mapped the hydrocarbon potential of known reservoirs but has also discovered previously untested structural zones, providing a fresh perspective on potential hydrocarbon accumulations.

The seismic interpretation has played a crucial role in unraveling the structural complexity of the Kukih Field. Growth faults, fault-assisted closures, and the presence of major and minor faults have been delineated, contributing to a comprehensive understanding of the field's trapping mechanisms. This structural information, combined with facies analysis, has shed light on the depositional context, indicating a channelized environment with characteristics of a prograding delta. The identification of Oil–Water-Contacts (OWC) and porosity analysis has further enhanced our understanding of reservoir dynamics. The challenges associated with mapping hydrocarbon-bearing formations through resistivity logs have been acknowledged, underscoring the necessity for advanced logging techniques.

Generally, the multidisciplinary approach employed in this study has yielded significant contributions in understanding the geological characteristics of the reservoir. In addition, it has introduced an innovative framework for making informed decisions in the field of hydrocarbon exploration. The outcomes of this research provide valuable guidance for upcoming exploration and production endeavors in the Onshore Niger Delta region, underscoring the significance of optimizing resource utilization and minimizing uncertainties in the development of oil and gas fields.
